# *De novo *protein sequence analysis of *Macaca mulatta*

**DOI:** 10.1186/1471-2164-8-270

**Published:** 2007-08-08

**Authors:** Nilesh S Tannu, Scott E Hemby

**Affiliations:** 1 Department of Physiology and Pharmacology, Wake Forest University School of Medicine, Winston-Salem, NC, USA

## Abstract

**Background:**

*Macaca mulatta *is one of the most utilized non-human primate species in biomedical research offering unique behavioral, neuroanatomical, and neurobiochemcial similarities to humans. This makes it a unique organism to model various diseases such as psychiatric and neurodegenerative illnesses while also providing insight into the complexities of the primate brain. A major obstacle in utilizing rhesus monkey models for human disease is the paucity of protein annotations for this species (~42,000 protein annotations) compared to 330,210 protein annotations for humans. The lack of available information limits the use of rhesus monkey for proteomic scale studies which rely heavily on database searches for protein identification. While characterization of proteins of interest from *Macaca mulatta *using the standard database search engines (e.g., MASCOT) can be accomplished, searches must be performed using a 'broad species database' which does not provide optimal confidence in protein annotation. Therefore, it becomes necessary to determine partial or complete amino acid sequences using either manual or automated *de novo *peptide sequence analysis methods.

**Results:**

The recently popularized MALDI-TOF-TOF mass spectrometer yields a complex MS/MS fragmentation pattern difficult to characterize by manual *de novo *sequencing method on a proteomics scale. Therefore, PEAKS assisted *de novo *sequencing was performed on nucleus accumbens cytosolic proteins from *Macaca mulatta*. The most abundant peptide fragments '*b-ions *and *y-ions*', the less abundant peptide fragments '*a-ions*' as well as the *immonium ions *were utilized to develop confident and complete peptide sequences *de novo *from MS/MS spectra. The generated sequences were used to perform homology searches to characterize the protein identification.

**Conclusion:**

The current study validates a robust method to confidently characterize the proteins from an incomplete sequence database of *Macaca mulatta*, using the PEAKS *de novo *sequencing software, facilitating the use of this animal model in various neuroproteomics studies.

## Background

Many species have been used to model various aspects of human diseases including mental illness. However, the complexity of human biochemistry, anatomy and behavioral factors are not easily modeled in all species and warrant the use of species which have greater degrees of functional equivalence for the measures under investigation. For example, the experimental use of *Macaca mulatta *(rhesus monkey) has been essential for expanding our knowledge of neurodevelopmental, neurodegenerative and organic human brain diseases, as well as, for normal brain function due in large part to close similarities in neuroanatomy, neurobiochemistry and behavior compared with other species. Moreover, the use of *Macaca mulatta *has significant translational value for understanding the influence of alterations in gene and protein expression in human disease processes.

One potential obstacle to comprehensive assessments of protein alterations is the relative paucity of available protein annotations for rhesus monkeys. Currently; NCBInr, Swiss-Prot and TrEMBL list 330210, 14991 and 55805 human protein annotations, respectively. However, NCBInr, Swiss-Prot and TrEMBL list only 41968, 297 and 1801 protein annotations for rhesus monkey, respectively. The highly uncharacterized nature of the rhesus monkey proteome makes it difficult to identify proteins, demonstrate differential regulation of proteins and investigate their post-translational modifications. The characterization of proteins of interest from rhesus monkey using the standard database search engines (e.g., MASCOT) has a limitation in that 'broad species database' searches are needed which results in less than optimal protein annotation. This limitation can be overcome in some respects using a *de novo *sequencing strategy, in which partial or complete amino acid sequence information is obtained using either manual or automated *de novo *peptide sequence analysis. This approach has been successfully utilized in recent studies to characterize peptides bound to class I MHC molecule HLA-A2.1[1], human skin elastin protein[2] and proteins from unsequenced genome of *Halorhodospira halophila*[3].

While manual protein sequencing via Edman degradation yields exact amino acid sequence without ambiguity, the procedure is laborious and does not lend itself to high-throughput analysis. It also lacks the sensitivity of mass spectrometry and can be halted by the presence of blocked amino acids. Fortunately, automated software tools have been developed to characterize the amino acid sequences generated from tandem mass spectrometry such as the complex MS/MS fragmentation pattern generated by MALDI-TOF-TOF mass spectrometer. Quality MS/MS spectrum consists of a ladder for *y-ions *and *b-ions *peaks. *De novo *sequencing uses the mass difference between two adjacent ions to deduce the peptide fragment sequence. However, factors such as incomplete fragmentation (whereby not all the *y- *and *b-ions *are present in the spectrum), imprecise precursor ion selection due to overlapping peptide fragment masses, low signal-to-noise ratio and unpredicted post-translational modifications (PTMs) complicate the manual *de novo *sequencing. *De novo *sequencing enables the analysis of quality MS/MS spectra which fails to generate protein identification after database searches, which is the case for the majority of proteins in rhesus monkeys. In fact, *de novo *sequencing is the only alternative for study of species with incomplete databases and databases which are not in the public domain [2,4-6]. Several strategies have been utilized more recently for *de novo *sequencing including chemical derivatization that add acidic, fixed charge or basic moieties[1,7-13]. These have included various derivatization protocols such as sulfonation of the peptide N-terminal group. This derivatization creates a strong acidic group which greatly enhances fragmentation ability of tryptic peptides to produce *b *and *y *fragments. The use of ^18^O incorporation at the C-terminus of peptides during protein hydrolysis has also been applied [14,15]. The current study shows the utility of underivatized peptides using MALDI-TOF-TOF.

To this end, *de novo *sequencing of peptides isolated from cytosolic fractions of the ventral striatum of rhesus monkeys was performed. The ventral striatum/nucleus accumbens is an integral component of the cortico-straital-palldial-thalamic/mesencephalic circuit which is involved in sensorimotor integration. Furthermore, dysregulation of the ventral striatum has been implicated in a variety of psychiatric disorders, including substance abuse [16,17], schizophrenia [18-20] and depression [21-23]. The use of the ventral striatum tissue and the cytosolic fraction from this region is based on the research interests of our laboratory. However, the *de novo *sequencing strategy presented here is generalizable to all brain regions, tissues and other protein preparations.

The presented method consists primarily of *de novo *sequencing of underivatized peptides using the MALDI-TOF-TOF, compared to various derivatizing strategies used recently for various *de novo *studies. The generated sequences were used to perform homology searches to characterize the protein identification. The current study validates a robust method to confidently characterize proteins from an incomplete sequence database of *Macaca mulatta *thereby facilitating the use of this animal model in various neuroproteomics studies.

## Results and discussion

**Figure 1:**
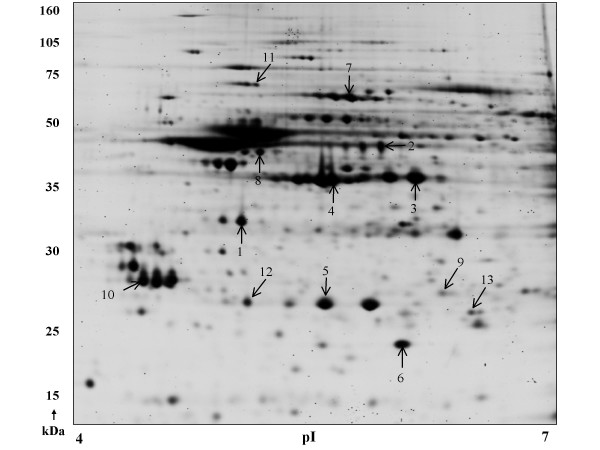
**Representative 2D gels of *Macaca mulatta *protein sample stained with SyproRuby™**. The polypeptide molecular mass scale in kDa is depicted on the y-axis while the x-axis shows the pI range. The proteins were resolved in 4–7 linear pH gradient (Immobiline DryStrips; 240 × 3 × 0.5) and 8–15% gradient SDS-PAGE (2400 × 2000 × 1 mm). The results of the proteins that were identified (indicated by arrows) by in-gel trypsin digestion and MALDI-TOF-TOF followed by de novo sequencing are elaborated in Table 1.

Comparison of the rhesus monkey *de novo *sequences with sequences in the human protein database enabled the validation of the *de novo *capabilities of the present method. This was accomplished by performing the detailed *de novo *method on tandem mass spectrometer spectra from *Ho*mo *sapiens *samples and objectively assessing the accuracy of the *de novo *sequencing by performing conventional database search (MASCOT) on the same spectra. Figure 1 shows the preliminary separation of proteins from nucleus accumbens of *Macaca mulatta *using the two-dimensional gel electrophoresis. The protein spots selected for *de novo *sequencing analysis ranged between 11- to 70-KDa and pI of 4.5–6.5 with a uniform distribution over the entire 2-D gel with respect to the *M*_r _and pI. The labeled protein spots were subjected to in-gel trypsin digestion after de-staining the gel plugs. The peptide fragments extracted from the gel plugs were then subjected to tandem-mass spectrometry using the ABI 4700 proteomics analyzer (MALDI-TOF-TOF).

**Figure 2:**
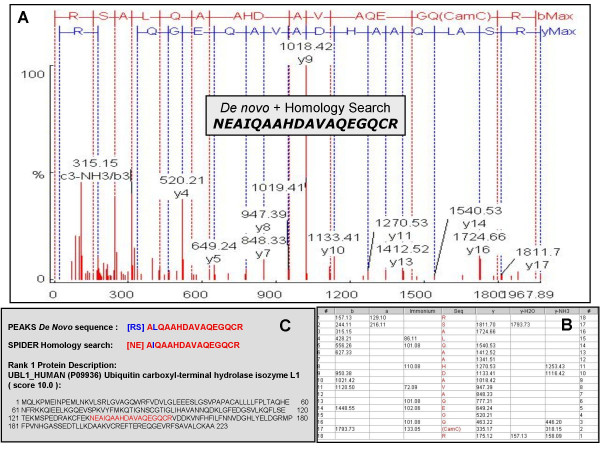
**Representative *De novo *analysis of a MALDI-TOF-TOF spectrum**. **(A) **The x- and y-axes show the mass to charge (*m/z*) ratio and the % abundance of the precursor ion fragments, respectively. The MS/MS spectrum was analyzed by PEAKS *de novo *sequencing software to generate '**RSALQAAHDAVAQEGQCR**'. **(B) **The table details peptide fragments '*b-ions*, *y-ions *and *a-ions*' and the neutral losses of water and ammonia for *b-ions *and *y-ions *as well as the *immonium ions *to develop confident and complete peptide sequence *de novo *from MS/MS spectrum. **(C) **The SPIDER homology search of this peptide resulted in sequence from Ubiquitin carboxyl-terminal hydrolase isozyme L1 as '**NEAIQAAHDAVAQEGQCR**'.

Tandem-mass spectra were then submitted for database searching (GPS Explorer: MASCOT), allowing to be searched with and without all known post-translational modifications, for protein characterization using the limited *Macaca mulatta *database. The majority of spectral analyses yielded no positive characterization, at which point spectra were subjected to PEAKS *de novo *analysis. According to the manufacturer, "the algorithm first computes a *y*-ion matching score and a *b*-ion matching score at each mass value according to the peaks around it. If there are no peaks around a mass value, a penalty value is assigned. The algorithm then efficiently computes many amino acid sequences that maximize the total scores at the mass values of *b*-ions and *y*-ions. These candidate sequences are further evaluated by a more accurate scoring function, which also considers other ion types such as immonium ions and internal-cleavage ions (Figure 2). The problem of ion absence is addressed because the PEAKS model assigns a score (or penalty) for each mass value. The software also computes a 'positional confidence' for each amino acid in the final result by examining the consensus of the top-scoring peptides" [4].

**Figure 3:**
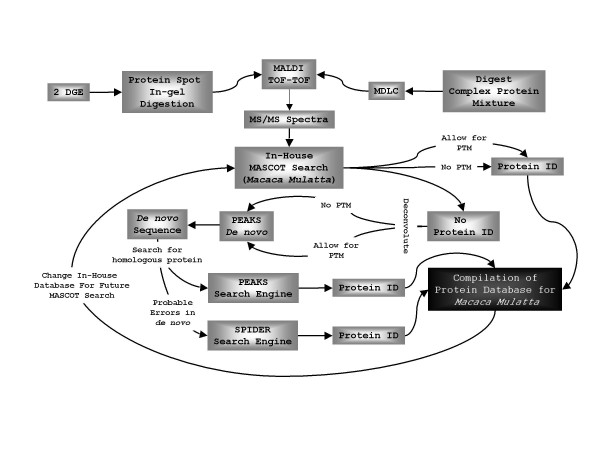
Schematic of the methodology for compilation of protein database for *Macaca mulatta *from *De Novo *analysis of MALDI-TOF-TOF spectra.

Thirteen targeted protein spots (Figure 1) were identified by MALDI-TOF-TOF followed by peptide sequencing using PEAKS Studio 4.0 *de novo *sequencing software. The generalized schematic of the methodology used in the current study to compile a database for *Macaca mulatta *is depicted in Figure 3. Detailed information of the confirmed protein characterization are elaborated in Table 1 with respect to the precursor mass, *m*/*z *error (ppm), PEAKS and SPIDER score for confidence interval (%) for the PEAKS *de novo *generated peptide sequences and their corresponding homology searches. This method characterized 13 protein spots out of 30 protein spots initially selected for *de novo *analysis.

**Table 1:** The proteins identified by *de novo *amino acid sequencing using MALDI-TOF-TOF

**#**	**Precursor ion (m/z)**	***De novo *peptide sequence**	**Homology search peptide sequence [PEAKS(◆)/SPIDER(●)]**	**Score %(◆) arbitrary(●)**	**Sequence assigned to protein (*Species*)**
1	1042.5265 1667.8766	LVTFYEDR VPAFLSAAEVEEHLR	LVTFYEDR(◆) VPAFLSAAEVEEHLR(◆)	97.81(◆) 99(◆)	Crystallin, mu (*Homo Sapiens*)
2	1631.7706	FQNIDFAEEVYTR	FQNIDFAEEVYTR(◆)	99(◆)	Guanine aminohydrolase(*Homo Sapiens*)
3	1303.7262	VLTPELYAELR GTGGVDTAAVGGVFDVSNADR	VLTPELYAELR(◆) GTGGVDTAAVGGVFDVSNADR (●)	99(◆) 12.3(●)	Brain creatine kinase (*Homo Sapiens*)
4	1516.6975 1954.0564	[*RT*]YDESGPS[*L*]VHR VAPEEHPVLLTEAPLNPK	[*QE*]YDESGPS[*I*]VHR(●) VAPEEHPVLLTEAPLNPK(●)	7.6(●) 11.2(●)	Actin beta (*Homo Sapiens*)
5	1967.8951 1648.6646	[*RS*]A[*L*]QAAHDAVAQEGQCR [*N*]GHLYELDGR	[*NE*]A[*I*]QAAHDAVAQEGQCR(●) [*D*]GHLYELDGR(●)	10.0(●) 5.8(●)	Ubiquitin carboxyl-terminal esterase L1 (*Homo Sapiens*)
6	1211.6733	Q [*L*]TVNDL[*VP*]GR EGGLGPLNIPLLADVTR	Q[*I*]TVNDL[*PV*]GR(●) EGGLGPLNIPLLADVTR(◆)	5.6(●) 95.97(◆)	Peroxiredoxin 2 isoform b (*Homo Sapiens*)
7	1684.9053 2560.2588	AAVEEGIVLGGGCALLR LVQDVANNTNEEAGDGTTTATVLAR	AAVEEGIVLGGGCALLR(◆) LVQDVANNTNEEAGDGTTTATVLAR(◆)	98.28(◆) 98.49(◆)	Heat shock 60 kD protein 1 (*Homo Sapiens*)
8	1987.0314	AIAELGIYPAVDPLDSTSR	AIAELGIYPAVDPLDSTSR(●)	11.2(●)	ATP synthase beta chain, mitochondrial precursor (*Homo Sapiens*)
9	1917.9341	FQDGDLTLYQSNTFLR	FQDGDLTLYQSNTFLR(◆)	96.24(◆)	GST pi enzyme (*Macaca mulatta*)
10	1256.55	NCSETQYERK	NCSETQYERK(◆)	99.1(◆)	14-3-3 gamma protein (*Homo Sapiens*)
11	1253.6162 1981.9982	FEELNADLFR TVTNAVVTVPAYFNDSQR	FEELNADLFR(◆) TVTNAVVTVPAYFNDSQR(◆)	97.95(◆) 99.49(◆)	Heat shock 70 kDa protein 8 isoform 1(*Homo Sapiens*)
12	1917.9427	SIQEIQELDKDDESLR	SIQEIQELDKDDESLR(◆)	99.17(◆)	Rho GDI alpha (*Homo Sapiens*)
13	1337.7222	PPYTVVYFPVR	PPYTVVYFPVR(◆)	98.1(◆)	Glutathione S-transferase

The tandem-mass spectra were analyzed by PEAKS *de novo *sequencing software to generate amino acid sequences (Figure 2). All tandem-mass spectra were deconvoluted to minimize the error in *de novo *sequencing. Figure 2 shows the fragmentation pattern of a precursor ion with m/z of 1967.8951. As has been documented previously and can be noted in the spectrum (Figure 2), complementary information is not always available for all *b-ions *and *y-ions *and not all the immonium ions are represented in the spectra. Spectral analysis is further complicated by the appearance of some *a-ions*, neutral losses of water and ammonia for *b-ions *and *y-ions*. These analysis caveats render the ability to obtain a manual *de novo *sequence tedious if not impossible. As elaborated in the Methods Section, the PEAKS *de novo *sequencing utilizes most abundant peptide fragments '*b-ions *and *y-ions*'; the less abundant peptide fragments '*a-ions*'; the neutral losses of water and ammonia for *b-ions *and *y-ions*; as well as the *immonium ions *to develop confident and complete peptide sequences *de novo *from MS/MS spectra[24]. The *b-*, *y-*, *a-*, and *immonium-ions *as well as the neutral losses of water and ammonia for *y-ions *are tabulated in Figure 2 for the amino-acid sequence 'RSALQAAHDAVAQEGQCR'. The tandem-mass spectrum in Figure 2 is representative of the similar analysis performed on the remaining protein spots from Figure 1.

Twenty peptide sequences were characterized by PEAKS *de novo *sequence analysis software from 13 protein spots (Table 1). The generated sequences were used to perform homology searches to characterize proteins. As a standard measure, all *de novo *generated amino-acid sequences were searched further for homologous sequences using the PEAKS homology search engine against the *Mammalian *database. Out of the twenty *de novo *generated sequences subjected to PEAKS homology search, thirteen yielded positive protein characterization (Table 1). All peptide sequences exhibited homology to *Homo sapiens*, with the exception of the sequence from one spot (GST pi enzyme: *Macaca mulatta*), The inability of the PEAKS homology search to resolve the remaining seven sequences may be attributed to the fact that the software assumes that the *de novo *sequence is 100% correct. Whereas standard BLAST assumes 100% accuracy of the *de novo *sequence, SPIDER software accounts for possible errors in *de novo *sequencing. Also, it should be noted that the conventional search engines such as BLAST and FASTA are designed to handle queries which are longer than 35 amino acids. Prototypically, the peptide sequences obtained after trypsin digestion are not longer than 10–15 amino acids. SPIDER software was utilized for homology based database searches in instances where PEAKS homology searches failed to provide positive protein identification. Such errors were characteristically due to partially correct sequence tags and replacement of an amino acid segment by another segment with approximately the same mass. The criteria used for the SPIDER based searches were as follows: non-gapped homology match; mass tolerance of 0.1 Da; NCBInr database; leucine equals isoleucine; lysine equals glutamine; carbamidomethylation and methionine in oxidized form. The approach yielded positive characterization of the remaining seven peptide sequences. Of these, five peptide sequences resulted in the characterization of three new proteins previously not characterized by the PEAKS homology search. The remaining two peptides correspond to previously identified proteins; however, the peptides represent new characterizations.

Table 1 also shows that the peptide sequences generated by the PEAKS *de novo *sequencing software returned identical sequences when searched for homologous sequences in the database, with the exception of four peptides belonging to three proteins. Thus, the PEAKS *de novo *sequencing software was able to provide positive protein identification in most instances. An example of the added benefit of coupling PEAKS *de novo *sequencing software with the SPIDER homology search is shown in Figure 2. The original sequence generated by the PEAKS *de novo *software was '[*RS*]A [*L*]QAAHDAVAQEGQCR', whereas the SPIDER homology search returned a sequence of '[*NE*]A [*I*]QAAHDAVAQEGQCR' with a unequivocal score of 10 and associated to protein ubiquitin carboxyl-terminal esterase L1 (*Homo Sapiens*). At least a part of the error of the PEAKS de novo software was due to the *I*/*L *ambiguity.

Thirteen targeted protein spots (Figure 1) were identified by MALDI-TOF-TOF followed by peptide sequencing using PEAKS Studio 4.0 *de novo *sequencing software (e.g. Figure 2). The generalized schematic of the methodology used in the current study to compile a database for *Macaca mulatta *is depicted in Figure 3. The detailed information of the confirmed protein characterization are elaborated in Table 1 with respect to the precursor mass, *m*/*z *error (ppm), PEAKS and SPIDER score for confidence interval (%) for the PEAKS *de novo *generated peptide sequences and their corresponding homology searches. This method characterized 13 protein spots out of 30 protein spots initially selected for *de novo *analysis.

## Conclusion

Contemporary proteomics requires prompt and confident protein identification of proteins of interest. The ability to utilize animal models to study the biochemical correlates of human disease requires a more complete database of those species as a prerequisite. To this end, the *de novo *sequencing strategy presented here provides a rapid and reliable means to identify proteins in *Macaca mulatta *– a species for which publicly available protein databases are very limited. However, it is important to note that this strategy is generalizable to other tissues, protein preparations and species and is not exclusive for *Macaca mulatta *or for cytosolic protein fractions from brain.

From 30 excised gel spots 13 were identified by mass spectrometry coupled with PEAKS *de novo *analysis software. Among the proteins were receptor-associated proteins, proteins involved in intra-cellular signaling, cytoskeletal structure, protein folding, hormonal changes and regulation of oxidative stress. The current study was undertaken to delineate a preliminary proteomics scale methodology to identify proteins *de novo *from NAc cytosol in the primate brain. Following mass spectrometric analysis, the most abundant peptides in the mixture led to the most accurate protein identification – hence, less abundant proteins may be overlooked. However, this caveat holds for all two dimensional gel-based proteomic approaches for studying disease states. Nevertheless, the present results provide the first preliminary *de novo *proteomic profile from *Macaca mulatta *and will form the basis of the future proteomics scale studies using non-human primate.

## Methods

### Subjects and tissue

Four adult male rhesus monkeys (*Macaca mulatta*) were restrained with Telazol, given intravenous heparin and then an overdose of intravenous sodium pentobarbital. After the confirmed absence of brain stem reflexes was established, the monkeys were transcardially perfuse with phosphate buffered saline to evacuate brain vasculature. Brains were blocked using a rhesus monkey brain matrix that allows 4 mm coronal blocks at various AP locations (Electron Microscopy Sciences, Ft. Washington, PA). Blocks were divided into their two component hemispheres – one for fresh frozen sections at -80°C and the other for dissection of blocks for paraffin embedding. Brain tissue from these monkeys was frozen within 40 minutes of the time of death. All experiments were conducted in accordance with the National Institutes for Health Guide for the Care and Use of Laboratory Animals.

### Protein isolation and fractionation

One hundred and fifty milligram punches were dissected from NAc from each subject. A steel mortar and pestle chilled in dry ice were used to pulverize the frozen brain tissue from each subject separately into a dry homogenate in the presence of liquid nitrogen. Tissue proteins from each subject were fractionated into membrane, nuclear and cytosolic fractions as described previously [25,26]. The tissues were homogenized in 10 mM HEPES, 10 mM NaCl, 1 mM KH_2_PO_4_, 5 mM NaHCO_3_, 1 mM CaCl_2_, 0.5 mM MgCl_2_, 5 mM EDTA, 1 mM phenylmethylsulfonylfluoride, 10 mM benzamidine, 10 μg/ml aprotinin, 10 μg/ml leupeptin, 1 μg/ml pepstatin. The tissue homogenate from each subject was centrifuged using a swinging bucket rotor (Beckman Coulter SW55Ti) at 5333 × g for 5 min. The supernatant from each subject (cytosolic and crude membrane fraction) was further centrifuged at 59,255 × g for 30 min at 4°C and the cytosolic supernatant was stored at -80°C.

### SDS-Polyacrylamide Gel Electrophoresis (PAGE)

The protein quantitation for SDS-PAGE was accomplished using the bicinchoninic acid protein assay kit (Pierce, Rockford, IL). Laemmli sample buffer was used to achieve equivalent protein concentrations for all samples. Thirty micrograms of protein from each sample was heated to 95°C for 5 min and electrophoresed on 10% Tris-HCl SDS-PAGE gels (BioRad). Gel fixation, staining by Pro-Q^® ^Diamond phospho-protein stain and the SyproRuby™ staining of the SDS-PAGE was completed as described in detail for 2D-PAGE.

### Extraction of proteins for 2D-PAGE

The cytosolic protein fraction from each subject was precipitated by 2-D clean-up kit (GE Healthcare) at 20°C overnight, pelleted by centrifugation (12,000 × g for 5 min) then air-dried for 2 min. Next, pellets were dissolved in re-hydration buffer [RB; 7 M urea, 2 M thiourea, 4% (w/v) 3-[(3-cholamidopropyl) dimethylammonio]-1-propane-sulfonate. The protein solution in RB was supplemented with immobilized pH gradient buffer 2% and 2% dithiothreitol (DTT) (10 mg/ml). The protein concentrations were established by the urea and detergent compatible 2D-Quant kit (Amersham Biosciences).

### 2D-PAGE

Two hundred microgram aliquots of cytosolic proteins from each subject were diluted in 400 μl of RB and increased to a final volume of 450 μl with destreak reagent (GE Healthcare). Immobiline™ DryStrips (240 × 3 × 0.5 mm, pH 4–7 linear) were re-hydrated for 10 hr on an Amersham Pharmacia Biotech IPGphor [27] followed by sequential isoelectric focusing (IEF) of samples as follows: 100 V for 100 V-hr, at 500 V for 500 V-hr, at 1000 V for 1000 V-hr, at 4000 V for 4000 V-hr, at 8000 V for 13500 V-hr (gradient) and at 8000 V for 60,000 V-hr. Fifty μA current was passed per strip maintaining the platform temperature at 20°C. Next, strips were equilibrated by adding a solution containing; 6 M urea, 1.5 M Tris-HCl, pH 8.8, 30% (v/v) glycerol, 2% (w/v) SDS and 2% (w/v) DTT and gentle rocking for 10 min, to reduce the disulfide bonds. Next, a solution containing 6 M urea, 1.5 M Tris-HCl, pH 8.8, 30% (v/v) glycerol, 2% (w/v) SDS and 2.5% (w/v) iodoacetamide was added to block sulphydryl groups.

After equilibration, the IPG strips were transferred to 2-D slab gels using 0.6% agarose stacking gel. The proteins were further separated on the basis of their molecular weight on 12.5% SDS-PAGE (2400 × 2000 × 1 mm) at a constant 4 W per gel until the dye front reached the end of the gel using the Ettan Dalt II System (GE Healthcare, Piscataway, NJ). Ten μl of the molecular mass marker (Amersham Rainbow marker RPN 800) was loaded on 2 mm^2 ^filter paper which was placed on the basic end of the IPG strip [28,29]. To visualize the proteome from this specific pH and mass range, gels were stained with Sypro Ruby™ stain overnight. The excess stain was removed by 10% methanol, 6% glacial acetic acid for 20 minutes. Protein spots of interest were subjected to MALDI-TOF-TOF [30].

### In-gel trypsin digestion

Individual protein spots from the 2-D gels were excised with 1.5 mm diameter gel cutter (The Gel Company, San Francisco, CA) [31]. The gel spots were washed for 20 min, twice in 100 μl of solution of 50 mM ammonium bicarbonate, 50% methanol (v/v) in distilled water and once in 75% acetonitrile in distilled water for 30 min or until the gel plugs turned opaque. Twenty micrograms of lyophilized trypsin (883 pmol; Promega, Madison, WI) was reconstituted in 1 ml of 20 mM ammonium bicarbonate and incubated for 15 min at 37°C. The gel fragments were dried by vacuum centrifugation and then incubated overnight with 10 μl (200 ng) of trypsin at 37°C. The supernatant from trypsin digest was transferred to a low retention 96-well plate. Peptides from the gel pieces were sequentially extracted twice in 100 μl of extraction buffer [50% (v/v) acetonitrile, 0.1% (v/v) trifluoroacetic acid (TFA) in distilled water (DW)]. The original tryptic supernatant and the supernatants from two sequential extractions were combined and dried in a vacuum centrifuge. The dried peptides from each gel plug were dissolved in 5 μl of 50% (v/v) acetonitrile, 0.1% trifluoroacetic acid in distilled water and 0.5 μl deposited on the stainless-steel MALDI target plate. After drying, the spot residue was mixed with 0.5 μl of 5 mg/ml of α-cyano-4-hydroxy-cinnamic acid (CHCA; Sigma-Aldrich, St. Louis, MO) in 50% (v/v) acetonitrile, 0.1% trifluoroacetic acid in distilled water[32].

### MALDI-TOF-TOF

Mass spectrometry analyses were performed using the Applied Biosystems 4700 Proteomics Analyzer (MALDI-TOF-TOF; Foster City, CA) in reflector mode for positive ion detection. The laser wavelength and the repetition rate were 355 nm and 200 Hz, respectively. All the MS spectra resulted from accumulation of at least 2000 laser shots. The peak detection criteria used were; minimum S/N of 8, local noise window width mass/charge (*m*/*z*) of 200 and minimum full-width half-maximum (bins) of 2.9. The mass spectra were calibrated using the three trypsin auto digest products: fragment 100–107 ([M + H]^+ ^= 842.51 Da), fragment 90–99 ([M + H]^+ ^= 1045.556 Da) and fragment 50–69 ([M + H]^+ ^= 2211.105 Da) [33]. A maximum of the ten strongest precursor ions per sample were chosen for tandem mass spectrometry (MS/MS) analysis. The following monoisotopic precursor selection were used for the MS/MS: minimum S/N filter of 10, excluding the most commonly observed peptide peaks for trypsin and keratin, and excluding the precursors within 150 resolution. In the TOF1 stage, all ions were accelerated to 1 kV under conditions promoting metastable fragmentation. The peak detection criteria used were; S/N of 8 and local noise window width of 250 (*m*/*z*).

### *De novo *sequencing

The PEAKS Studio 4.0 (Bioinformatics Solutions, Waterloo, Ontario, Canada) *de novo *sequencing software was used for automated *de novo *sequencing followed by manual confirmation of most sequences generated. A parent- and fragment-mass error tolerance of 0.08 u; trypsin as the protease with one maximum missed cleavage allowed; deconvolute the charge state in the spectra to generate a spectra in which each monoisotopic peak becomes singly charged; partial modification of cysteine (carbamidomethyl-cysteine) and methionine (oxidized), were used as the *de novo *sequencing parameters. The most abundant peptide fragments '*b-ions *and *y-ions*'; the less abundant peptide fragments '*a-ions*'; the neutral losses of water and ammonia for *b-ions *and *y-ions*; as well as the *immonium ions *were utilized to develop confident and complete peptide sequences *de novo *from MS/MS spectra [24]. Since the *Macaca mulatta *has an incomplete protein database, the sequences generated from each spectrum were used for protein identification by sequence homology in the mammalian database using either the PEAKS or SPIDER software (Software Protein Identifier). Therefore, the SPIDER software was useful when the *de novo *sequencing gave partially correct sequence tags and at instances where a segment of amino acids was replaced by another segment with approximately same masses [Han, Y JBCB; (3) 2005; 697–716]. The algorithm used for determining the probability based scoring with a given mass spectrum is described in detail by Ma B *et al *[24]. Protein identification was confirmed by checking the protein mass and pI accuracy [30].

## Abbreviations

NAc; nucleus accumbens, 2 DGE; two dimensional gel electrophoresis, 2-D; two dimensional, MALDI-TOF-TOF; matrix assisted laser desorption ionization-time of flight-time of flight, MS; mass spectrometry, MS/MS; tandem mass spectrometry PAGE; Polyacrylamide Gel Electrophoresis, RB; re-hydration buffer, IEF; isoelectric focusing, DTT; dithiothreitol, *m*/*z*; mass/charge, PMF; Peptide mass fingerprint

## Authors' contributions

The studies were conducted in the lab of SEH. SEH procured and dissected the tissue, isolated the cytosolic fractions and participated in the writing of the manuscript. NST conceived the study and its design. NST carried out the sample preparation and 2D-PAGE; produced and analyzed MALDI-TOF-TOF data; performed *de novo *sequencing and drafted the manuscript. Both the authors read and approved the final manuscript.
